# Alterations in the coupling functions between cortical and cardio-respiratory oscillations due to anaesthesia with propofol and sevoflurane

**DOI:** 10.1098/rsta.2015.0186

**Published:** 2016-05-13

**Authors:** Tomislav Stankovski, Spase Petkoski, Johan Raeder, Andrew F. Smith, Peter V. E. McClintock, Aneta Stefanovska

**Affiliations:** 1 Department of Physics, Lancaster University, Lancaster LA1 4YB, UK; 2 Faculty of Medicine, Ss. Cyril and Methodius University, 50 Divizija 6, Skopje 1000, Macedonia; 3 Institut de Neurosciences des Systèmes UMR_S 1106, Aix-Marseille Université, Marseille 13005, France; 4 Department of Anaesthesiology, Oslo University Hospital, Oslo 0424, Norway; 5 Department of Anaesthesia, Royal Lancaster Infirmary, Lancaster LA1 4RP, UK

**Keywords:** coupling function, cross-frequency coupling, anaesthesia, propofol, sevoflurane, brain–heart–respiration interactions

## Abstract

The precise mechanisms underlying general anaesthesia pose important and still open questions. To address them, we have studied anaesthesia induced by the widely used (intravenous) propofol and (inhalational) sevoflurane anaesthetics, computing cross-frequency coupling functions between neuronal, cardiac and respiratory oscillations in order to determine their mutual interactions. The phase domain coupling function reveals the form of the function defining the mechanism of an interaction, as well as its coupling strength. Using a method based on dynamical Bayesian inference, we have thus identified and analysed the coupling functions for six relationships. By quantitative assessment of the forms and strengths of the couplings, we have revealed how these relationships are altered by anaesthesia, also showing that some of them are differently affected by propofol and sevoflurane. These findings, together with the novel coupling function analysis, offer a new direction in the assessment of general anaesthesia and neurophysiological interactions, in general.

## 1. Introduction

General anaesthesia plays a crucial role in many surgical procedures, and it therefore has an enormous impact on human health. It is a drug-induced, reversible state characterized by unconsciousness, anti-nociception or analgesia, immobility and amnesia [1,2]. On rare occasions, however, the patient can remain unconscious longer than intended, or may regain awareness during surgery. There are no precise measures for maintaining the correct dose of anaesthetic, and there is currently no fully reliable instrument to monitor depth of anaesthesia. Although a number of devices for monitoring brain function or sympathetic output are commercially available [3], the anaesthetist also relies on clinical assessment and experience to judge anaesthetic depth. The undesirable consequences of overdose or unintended awareness might in principle be ameliorated by improved control if we could understand better the changes in function that occur during general anaesthesia, in particular the dynamical brain states, the dynamics of cardiovascular oscillations and their mutual interactions [4].

General anaesthesia can be induced by different anaesthetics which can affect different physiological regions, receptors and channels [5,6]. In this study, we used two of the most widely used anaesthetics—*propofol* and *sevoflurane*, i.e. we used one of the two in each anaesthesia measurement. Propofol is introduced intravenously, while sevoflurane is a sweet-smelling, non-flammable type of ether that is inhaled [7–10].

The central enigma in general anaesthesia is the nature of the unconscious state mediated in the brain. Neuronal states often manifest themselves as changes in brain electrophysiological activity, which emanates from the dynamics of large-scale cell ensembles oscillating synchronously [11,12] within characteristic frequency intervals. Individual ensembles communicate to integrate their local information flows into a common brain network. One way to describe such an integration or communication is through cross-frequency coupling, a method that has led to numerous studies elucidating the respective roles of cognition, attention, memory and anaesthesia [9,10,13–15]. Jirsa & Müller [13] recently identified different types of cross-frequency coupling based on use of the *power, phase or frequency* domains; in what follows, we focus on phase–phase cross-frequency couplings. Unlike earlier cross-frequency coupling methods, the approach that we will discuss assesses neuronal states through the computation of *coupling functions* describing the functional forms of individual cross-frequency interactions.

Coupling functions prescribe the physical rule specifying how the inter-oscillator interactions occur. They determine the possibility of qualitative transitions between the oscillations, e.g. routes into and out of phase synchronization [16]. Their decomposition can describe the functional contribution from each separate subsystem within a single coupling relationship. In this way, coupling functions offer a unique means of describing mechanisms in a unified and mathematically precise way. It is a fast growing field of research, with much recent progress on the theory [17,18] and especially towards being able to extract and reconstruct the coupling functions between interacting oscillations from data, leading to useful applications in cardiorespiratory interactions [19–21], chemistry [16], mechanics [22] and communications [23]. We will show that, in neuronal analysis, the cross-frequency coupling function describes much more than just a new way of measuring effects: it opens up a whole new perspective on the functional mechanisms underlying the functionality of the brain network.

The oscillatory processes of the brain are not only individually important to the function of the central nervous system, but they can also interact, both mutually and with other physiological oscillations. The latter comprise, e.g., the oscillatory processes of the cardiovascular system [24] including the heart and the lungs, which are closely associated because, working together, they provide the blood supply with oxygen and nutrients for the whole body including the brain. The brain's functional state is obviously of crucial importance in general anaesthesia and as such it provides the basis for number of measures [3] (including, e.g., the BIS (bispectral index) monitor by Medtronic (formerly aspect medical), the Entropy monitor by GE Healthcare, the Narcotrend index by MonitorTechnik, and others). However, although traditional anaesthetic monitoring includes only the on–off awake versus unconscious classification, indirect or surrogate measures of brain function, such as movement, blood pressure, heart rate, sweating and other anaesthesia-induced changes to the cardiovascular system [25–29], also provide valuable indicators [1]. Moreover, the two systems are connected in many ways, and some signatures of causal interaction have already been demonstrated [15]. For a comprehensive assessment of general anaesthesia one should therefore add a consideration of the (complex) interactions between the cardiovascular and brain oscillations [4], and the integration of their functions into what are interconnected physiological networks [4,15,30]. One may thus investigate the mechanisms and connections between the brain and the loss of consciousness [1,2] on the one hand, and, on the other hand, the cardiovascular system which is closely related to the function of the autonomous nervous system including anti-nociception, analgesia and the perception of pain [31–34].

**Figure 1.**
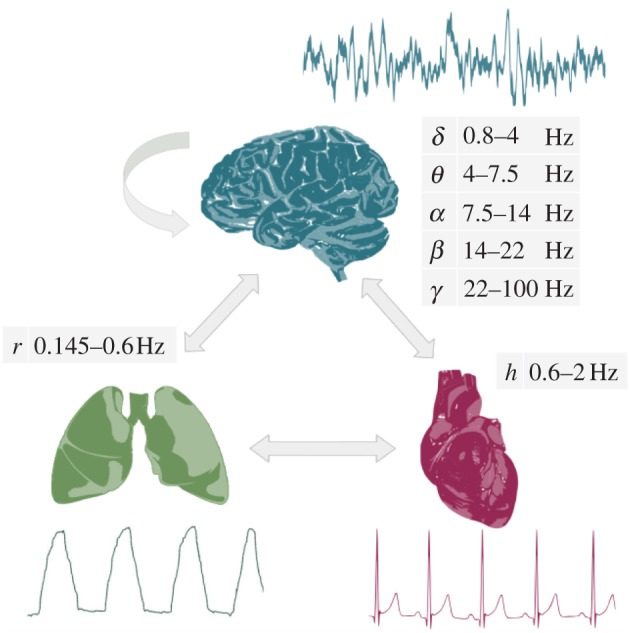
Schematic of the main aims of the study. We seek to investigate the interactions between oscillatory processes in the brain, lungs and heart, and to establish how they are affected by general anaesthesia. The interactions are assessed by reconstruction of the coupling functions. The analyses are performed on non-invasive measurements of the electroencephalogram (EEG), the respiration signal from expansion of the thorax and the electrocardiogram (ECG). Samples of raw measurements are shown adjacent to each of the organs, as are also the relevant cross-frequency intervals. (Online version in colour.)

In this paper, we seek to establish the functional laws defining the mutual interactions between the brain, heart and the lungs (figure 1) in general anaesthesia. The study is based on three complementary pillars: (i) anaesthesia with two of the most widely used anaesthetics, using the same experimental set-up; (ii) application of the novel methodology of cross-frequency *coupling functions* to determine phase-causal links and to probe the interaction mechanisms directly, and (iii) assessment of general anaesthesia based on the combined dynamics and interactions of the *brain, lungs and heart* oscillations.

## 2. Material and methods

### (a) Inference of cross-frequency coupling functions

Cross-frequency couplings are usually inferred by methods based on the statistics of the coupled signals, such as the correlation and (bi-) coherence measures. Such approaches tell one about the *functional connectivity* [35], but they do not provide information about causality or about the form of the coupling functions. By contrast, however, we now show that inference of cross-frequency couplings based on a model of coupled phase oscillators [36,37] and dynamical Bayesian inference [19,38,39] enables us to infer the *effective connectivity* [35], i.e. to estimate the coupling functions and the underlying causality. We note that the effective connectivity was initially discussed in relation to the spatial segregation of brain functions [40] and is often used in this sense by the neuroscience community. In this work, we do not study spatial connectivity but, rather, we exploit the mathematical concept of effective connectivity in determining the influence that one oscillator exerts on another, under a particular model of causal dynamics [35,41]. With its ability to infer time-evolving coupled dynamics in the presence of noise, dynamical Bayesian inference is ideal for the calculation of effective connectivity from neuronal oscillations.

The signals derived from the chosen cross-frequency intervals are oscillatory, and their interactions can be studied effectively through their phase dynamics. We therefore consider a model of two coupled phase oscillators [36] described by the stochastic differential equation
2.1
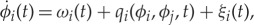

with *i*≠*j* for *i*,*j*={1,2} and where *ω*_*i*_(*t*) is the parameter for the natural frequency. The deterministic part given by the base functions *q*_*i*_(*ϕ*_*i*_,*ϕ*_*j*_,*t*) describes the self and the interacting dynamics. The external stochastic dynamics *ξ*_*i*_(*t*) is considered to be Gaussian white noise 〈*ξ*_*i*_(*t*)*ξ*_*j*_(*τ*)〉=*δ*(*t*−*τ*)*D*_*ij*_. Owing to the periodic nature of the deterministic dynamics, the base functions can be decomposed into infinite Fourier series 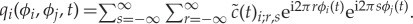. In practice, however, the dynamics is well described by a finite number of Fourier terms, so that one can rewrite the phase dynamics as 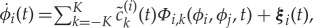, where 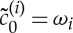, and the rest of *Φ*_*i*,*k*_ and 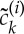 are the *K* most important Fourier components. The Fourier components *Φ*_*i*,*k*_ act as base functions for the dynamical Bayesian inference, through which the parameters 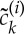 are evaluated. In the analysis, we used a second-order Fourier expansion (*K*=2). Two phase time-series and the order of expansion *K* act as inputs for the phase model which is inferred for each interaction (e.g. *δ*–*α*), from each subject.

Dynamical Bayesian inference [19,39] enables us to evaluate the model parameters 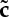, which give the time-evolving coupling functions and coupling strength in the presence of noise. From Bayes' theorem one can derive the minus log-likelihood function, which is of quadratic form. Assuming that the parameters are represented as a multivariate normal distribution (with mean 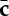, and covariance matrix ***Σ***≡*Ξ*^−1^), and given such a distribution for the prior knowledge using the likelihood function, one can calculate recursively [19] the posterior distribution of the parameters 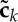 using only the following four equations:
2.2
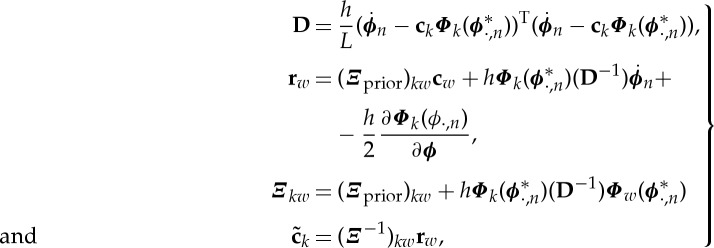

where summation over *n*=1,…,*N* is assumed, and summation over repeated indices *k* and *w* is implicit. We used informative priors and a special procedure for the propagation of information between consecutive data windows [19], which permitted the inference parameters that varied with time (for implementation and usage, see [42,43]). Given its ability to infer time-varying and noisy dynamics, our Bayesian method is especially well fitted for applications to EEG, ECG and respiration signals. A block diagram summarising the analysis procedure is provided in the electronic supplementary material.

Once we have the inferred parameters 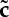, we can calculate the coupling quantities and characteristics. The coupling functions are evaluated on a 2*π*×2*π* grid using the relevant base functions, i.e. Fourier components scaled by their inferred coupling parameters. The coupling strength is calculated as the Euclidean norm of the inferred parameters for a particular coupling [42]. The correlation *ρ* of the coupling parameters from two coupling functions gives the similarity of the forms of the coupling functions, irrespective of amplitude [20]. All coupling characteristics can be evaluated either for the net coupling, or for individual coupling components.

### (b) Coupling decomposition model

**Figure 2.**
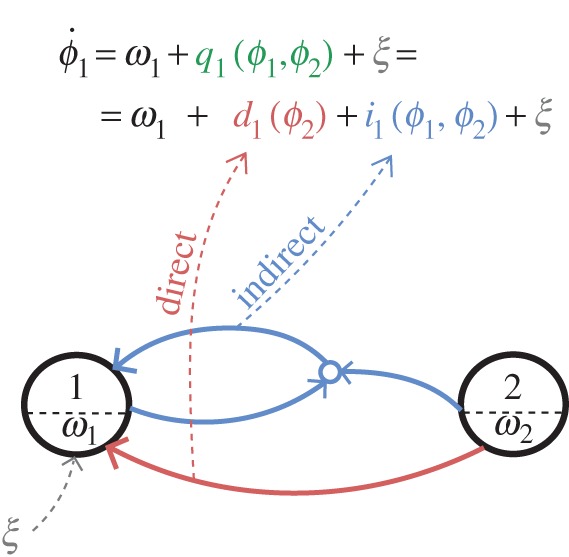
Model of the coupling decomposition. The dynamical equation (top) represents how one phase oscillator (index 1) is influenced by another (index 2). The net coupling *q*_1_(*ϕ*_1_,*ϕ*_2_) is decomposed into two functional entities: the direct *d*_1_(*ϕ*_2_) and the indirect *i*_*i*_(*ϕ*_1_,*ϕ*_2_), coupling functions. The dynamics is also characterized by a natural frequency parameter *ω*_1_ and external noise perturbations *ξ*. (Online version in colour.)

The form of a coupling function depends on the differing contributions from individual oscillations. Changes in form may depend predominantly on only one of the phases (along one-axis), or they may depend on both phases, often resulting in a complicated and intuitively unclear dependance. This demonstrates the need for a model able to distinguish the individual functional contributions to a coupling. Accordingly, following the cardiorespiratory model [21], we present a generalized *coupling decomposition model* (figure 2).

Previous coupling treatments, including the cross-frequency coupling in neuroscience, have focused on the net coupling in one direction. Instead, we decompose the net coupling into two components depending on their functional roles: the direct and the indirect couplings (figure 2). Direct-coupling describes the influence of the direct (unidirectional) driving that one oscillator exerts on the other. Arguably, it is the most studied interaction in physiology, often linked to modulation mechanisms. We will see that direct-coupling is the dominant mechanism in most of the coupling functions. The second component, indirect-coupling, often called common-coupling, depends on the shared contributions of the two oscillators. The indirect coupling includes also the diffusive coupling given with the phase difference terms. The mechanism behind this coupling component (the small circle on the arrow in figure 2) can lie in some functional dependence from both of the current phase states, or it can be induced by a third system or process. Although we present the model in relation to phase dynamics, a similar functional decomposition of the couplings can also be applied to amplitude state dynamics.

In terms of the general theory of phase dynamics [36] (and equation (2.1)), the coupling function *q*_1_(*ϕ*_1_,*ϕ*_2_) can be expressed as the product of two functions
2.3
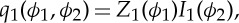

where *Z*_1_(*ϕ*_1_) is the phase response curve (PRC) of the first oscillator and shows how it responds to external perturbations, while *I*_1_(*ϕ*_2_) is the perturbation function through which the second oscillator acts on the first one. (The perturbation function is often given in a more general form like *I*_1_(*ϕ*_1_,*ϕ*_2_) [36]). In terms of equation (2.3), the direct-coupling component results from the existence of the constant part of the PRC *Z*_1_(*ϕ*_1_), while the common or indirect-coupling component results from the existence of the phase-dependent part of the PRC *Z*_1_(*ϕ*_1_) and the perturbation function *I*_1_(*ϕ*_2_).

### (c) Subjects and protocol

We measured 25 awake and 29 anaesthetized healthy subjects, aged 18–60 years, who were about to undergo elective surgery, all of whom had given their informed consent in writing. The research was approved by the relevant research ethics committees in Norway and the UK. Of 29 anaesthetized subjects, 14 were anaesthetized with propofol and 15 with sevoflurane.

There are two sets of recordings for every subject: the first while the subject was *awake* and resting, and the second while anaesthetized with either *propofol* or *sevoflurane* by random choice. Propofol anaesthesia was induced by infusing propofol until a plasma target concentration of 6.0 μgm l^−1^ was reached [44]. A laryngeal mask airway was inserted 2 min after the start of the infusion. After insertion, the target concentration was reduced to 3.0 μgm l^−1^ and the infusion was maintained at this rate throughout the measurement period. Some of the propofol patients became restless during induction (while unconscious) and eight of them were given a small dose (50–100 μg) of the very short-lived (*T*_half-life_≃4 min.) opioid remifentanil during induction. Owing to the small dose and the short half-life, this would not have significantly affected the signals. The other group of subjects were asked to breathe 8% sevoflurane through a close-fitting facemask until an end-tidal concentration of 5% was reached. A laryngeal mask airway was inserted, and then the sevoflurane turned off until the end-tidal concentration fell to 2%. The sevoflurane was then reinstituted to maintain the end-tidal concentration at 2% throughout the measurement period. After a further stabilization period, the anaesthetized set of signal recordings took place. Subjects breathed spontaneously during both sets of recordings. The BIS EEG electrode was placed frontally on the forehead (similar to the FP1 electrode from the 10–20 international system). All data were recorded simultaneously using a *Cardio & Brain Signals* signal conditioning system (Jožef Stefan Institute, Ljubljana, Slovenia) specially designed for the *BRACCIA* study. Following 24-bit A/D conversion at 1200 Hz, the signals were stored on a computer for subsequent analysis. They included the three-lead ECG, and the respiration signal measured with a thorax-belt, as well as the frontal EEG signal. All were of 22–32 min duration. The analyses were performed on equal-length segments of 20 min.

### (d) Signal processing and statistical analysis

The signals were first inspected visually, followed by automated artefact removal by interpolation. Data from subjects whose signals had many artefacts were disregarded and not analysed. The cross-frequency intervals were estimated by standard digital filtering procedures, including a FIR filter followed by a zero-phase digital filtering procedure (filtfilt in Matlab) to ensure that no time or phase lags were introduced by the filtering. The boundaries of the intervals extracted from the EEG signal were *δ*=0.8–4 Hz, *θ*=4–7.5 Hz, *α*=7.5–14 Hz, *β*=14–22 Hz and *γ*=22–100 Hz; the interval extracted from the respiration signal was *r*=0.145–0.6 Hz; and the extraction of the heart activity from the ECG signal was *h*=0.6–2 Hz. Wavelet power and coherence analyses, together with further clinical interpretation, will be presented elsewhere. For the EEG oscillations special care was taken in dealing with frequency spillage between intervals, heart artefacts and powerline artefacts [45].

The cardiac activity has been widely studied through heart rate variability (HRV) analysis [46]. Usually, the HRV signal is constructed by interpolation of the times of the R-peaks marked on an ECG signal, whence the variations in heart rate can be obtained up to a frequency of approximately 0.5 Hz, i.e. up to half of the main (fundamental) cardiac oscillation frequency at approximately 1 Hz. In this study, we focused on the coupled-oscillator approach [36,47], which meant that we required the cardiac main oscillation mode at approximately 1 Hz, which would of course get lost in an HRV estimation. By contrast, by band-filtration of the signal in the interval *h*=0.6–2 Hz around the main oscillation we were able to obtain well-defined phase estimates with intra-cycle resolution. Hence, we could analyse the phase interactions of the cardiac main oscillation mode, as required; we note, however, that this procedure would have led to the loss of some of the HRV variations, and especially those at the lower frequencies.

The phases of the filtered signals were estimated by use of the Hilbert transform [48], and the protophase-to-phase transformation [22] was then applied to the resultant protophases to obtain invariant observable-independent phases. To determine whether the coupling strength and coupling functions were not genuine, i.e. whether they happened by chance, the coupling of each of the relationships investigated was tested against intra- and intersubject surrogates [49]: the former were generated by randomizing the phase signals, and the latter by taking one of the phases from a different subject. In this way, the surrogates should be independent and any apparent coupling from the surrogate phases should be very low. From the large number of investigated relationships, only those exhibiting a statistically significant difference compared with their corresponding surrogates are discussed in the study. Similarly, for simplicity we present only the coupling in the predominant direction because that in the weaker direction was usually insignificant. To assess the statistical difference between groups of awake, propofol- and sevoflurane-anaesthetized subjects (and because of the non-normal distributions), we used the Wilcoxon statistical test, with *p*<0.05 considered as significant. The couplings were assessed independently; they did not form a statistical family and multiple comparison tests were not used. To present visually the differences between the distributions, we used standard boxplots which refer to the descriptive statistics (median, quartiles, maximum and minimum).

## 3. Results

### (a) Cross-frequency coupling functions

**Figure 3.**
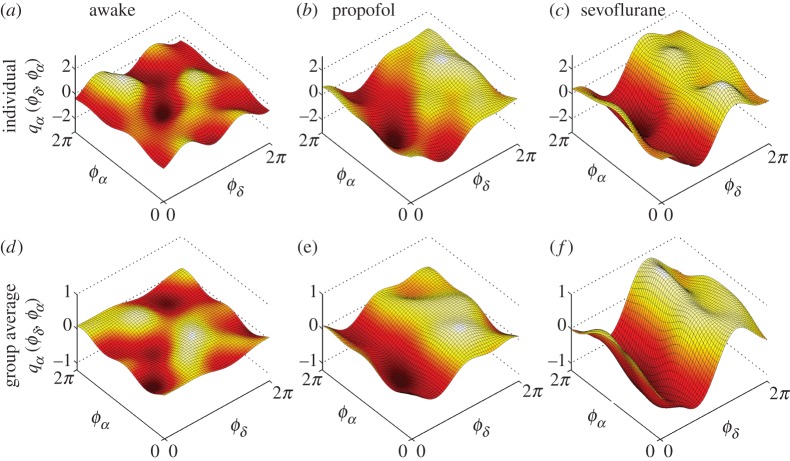
Cross-frequency coupling functions between *δ* and *α* brain oscillations. Each *δ*-to-*α* coupling function *q*_*α*_(*ϕ*_*δ*_,*ϕ*_*α*_) is evaluated from the *α*-dynamics and depends on the bivariate (*ϕ*_*δ*_,*ϕ*_*α*_) phases. (*a*–*c*) The coupling functions for one individual subject, while (*d*–*f*) the average coupling functions from all subjects within the group. Note that for comparison the vertical scale of coupling amplitude is shown on same interval for (*a*–*c*), and then for (*d*–*f*). Here, and throughout, we refer to *Awake* as the state when the subject is awake and resting; and *Propofol* and *Sevoflurane* when the subject is anaesthetized with propofol or sevoflurane, respectively. (Online version in colour.)

The application of dynamical Bayesian inference to bivariate phase signals leads to the parameters of the coupled phase model, from which the coupling functions can then be reconstructed. For clarity, we first present in detail the coupling function for one relationship only—the delta–alpha coupling figure 3. Examples of the delta–alpha coupling function for single representative subjects in their awake and anaesthetized states are shown in figure 3*a*–*c*. In the averaged coupling function for all subjects (figure 3*d*–*f*) the inter-subject variations are averaged out, and the remaining coupling function signifies a functional form that represents a deterministic law for all of the subjects.

Comparison of the coupling function shapes for individual subjects (figure 3*a*–*c*) with the corresponding averages over all subjects (figure 3*d*–*f*) reveals considerable similarity between subjects. The coupling functions for awake resting [50], propofol and sevoflurane (figure 3*a*–*f*) are, however, quite different from each other, both in the form and strength of the coupling. The delta–alpha coupling function for the awake state has a relatively complex and varying form, and low amplitude. The coupling functions for propofol and sevoflurane are similar and they look significantly different from those for the awake state. The sevoflurane coupling function has the largest coupling amplitude. The qualitative form of the delta–alpha coupling function (figure 3*f*) has a sine-like wave form along the *ϕ*_*δ*_-axis, while it is nearly constant along the *ϕ*_*α*_-axis. This strongly implies that much of the delta–alpha coupling comes from the direct contribution of the delta oscillation. The specific form of the delta–alpha coupling function (e.g. figure 3*f*) reveals the underlying functional coupling mechanism, i.e. it shows that, when the delta oscillations are between *π* and 2*π*, the sine-wave coupling function is higher and the delta activity accelerates the alpha oscillations; similarly, when the delta oscillations are between 0 and *π*, the coupling function is decreased and delta decelerates the alpha oscillations.

**Figure 4.**
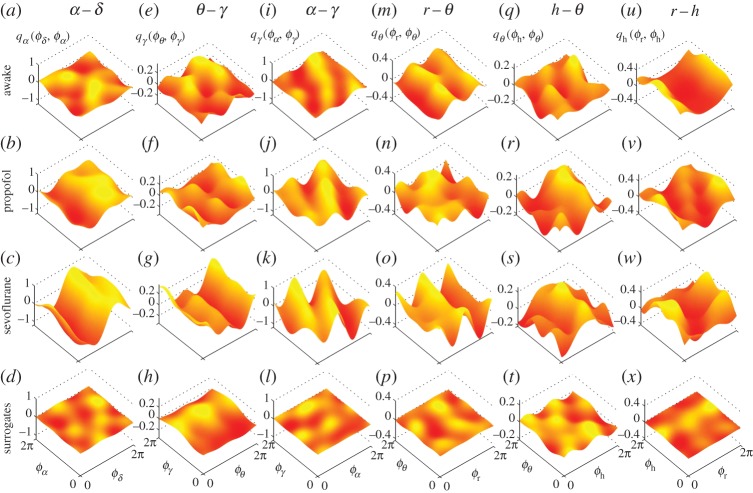
Cross-frequency coupling functions between neuronal and cardiorespiratory oscillations: (*a*–*d*) *δ*–*α*; (*e*–*h*) *θ*–*γ*; (*i*–*l*) *α*–*γ*; (*m*–*p*) *r*–*θ*; (*q*–*t*) *h*–*θ* and (*u*–*x*) *r*–*h*. The coupling functions are arranged in columns, and the states and surrogates are aligned horizontally. The coupling functions shown are the average over all subjects within a group and the vertical coupling scales are the same for each state within a relationship. The notation and interpretation of the individual coupling functions are the same as in figure 3. (Online version in colour.)

In figure 4, we summarize our results for the coupling functions of all significant coupling relationships. They include the cross-frequency coupling functions that emerge within the brain, and between the brain, the lungs and the heart oscillations (figures with enhanced resolution are provided in the electronic supplementary material). The delta–alpha relationship is presented again for completeness and comparison. The theta–gamma coupling functions (figure 4*e*–*h*) have different forms, depending on the state of awakeness, with propofol and sevoflurane taking similar forms. The coupling amplitude of the propofol theta–gamma coupling (figure 4*f*) is lowest. The form of the functions looks like a second-order sine wave which changes predominantly along the *ϕ*_*θ*_-axis. The alpha–gamma coupling functions (figure 4*i*–*l*) are of similar form, but their coupling amplitudes increase in anaesthesia, with the sevoflurane coupling function again being the highest. Interestingly, the qualitative form of the functions changes along both axes. This implies that the alpha–gamma coupling depends on both of the oscillations (alpha and gamma), or on the same indirect influence that affects them both.

The influence of respiration on brain theta oscillations is shown in figure 4*m*–*p*. There exist similarities in the form of the coupling functions between the awake and sevoflurane states, while the form of the propofol function seems qualitatively different. The direct influence of the phase *ϕ*_*r*_ of respiration is dominant in the awake and sevoflurane coupling functions. The coupling of the heart to theta oscillations is weak with a less-stable and time-varying form (figure 4*q*–*t*). The two anaesthetized heart–theta coupling functions are of similar form and are stronger than in the awake state. The strong coupling function between respiration and the heart oscillations (figure 4*u*–*x*) is the only one to have been studied previously, and our results confirm the earlier work [19–21]. More importantly, the propofol and sevoflurane anaesthesia made the form of the cardiorespiratory coupling function more time-varying and unstable—which is opposite to the effect of anaesthesia on the delta–alpha coupling (cf. figure 4*a*–*d*).

### (b) Effect of anaesthesia on the coupling strength

In order to assess the influence of anaesthesia, we first quantify the coupling (amplitude) strength. The latter has been extensively studied in earlier work [13,15,45,51]: wherever reference was made to coupling causality and directionality, it was in fact the net coupling strength, or a measure proportional to it, that was being evaluated. Our coupling decomposition enables us to go beyond this by quantifying the coupling strengths of the individual components of the net coupling.

**Figure 5.**
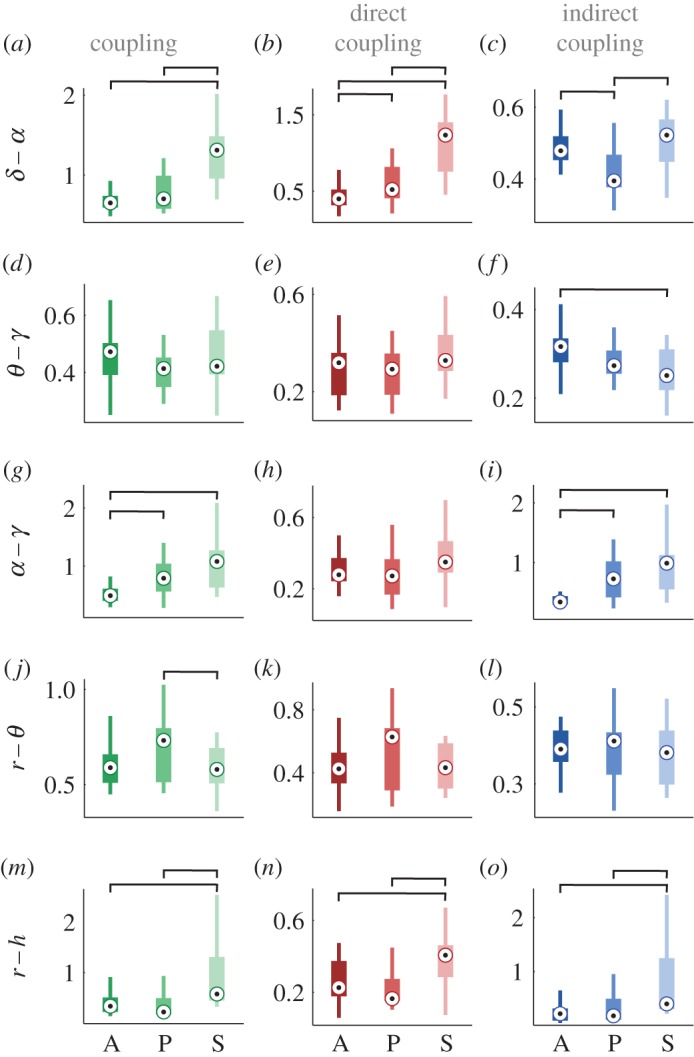
Anaesthesia-induced changes in coupling strength. Each boxplot shows the coupling strength distribution of a specific coupling relationship for the awakeness state indicated by the letter A (awake), P (propofol) or S (sevoflurane) on the abscissa. The coupling relationships are shown on the vertical axes, with each interaction as a separate row, including *δ*–*α* shown in (*a*–*c*), *θ*–*γ* (*d*–*f*), *α*–*γ* (*g*–*i*), *r*–*θ* (*j*–*l*) and *r*–*h* (*m*–*o*). The *h*–*θ* row is omitted because there were no significant changes. The columns correspond to the net, direct and indirect coupling components, respectively. The line connectors on the tops of individual panels indicate cases where the difference between two boxplot distributions was statistically significant (for statistical procedures see §2d). (Online version in colour.)

In figure 5, we summarize the changes of coupling strength induced by anaesthesia. The different effect on the separate coupling components is evident in the delta–alpha relationships shown in the top row of figure 5*a*–*c*. The net coupling with sevoflurane is significantly different from the awake and propofol states (figure 5*a*); for direct coupling all the states are different (figure 5*b*), while the indirect coupling for propofol was significantly the smallest (figure 5*c*). Note also that direct coupling is the dominant component of the net coupling. For the theta–gamma interaction, it is only the indirect coupling that differs between the awake and sevoflurane states (figure 5*f*). Anaesthesia increased significantly the net and indirect coupling strengths in alpha–gamma (figure 5*g*–*i*). This coupling is mostly defined by the indirect coupling component. The respiration–theta net coupling differed slightly between the two anaesthetics (figure 5*j*). Sevoflurane anaesthesia induced the strongest cardiorespiratory coupling strength, and this difference compared with other states is significant for all coupling types (figure 5*m*–*o*).

### (c) The effect of anaesthesia on the form of coupling functions.

**Figure 6.**
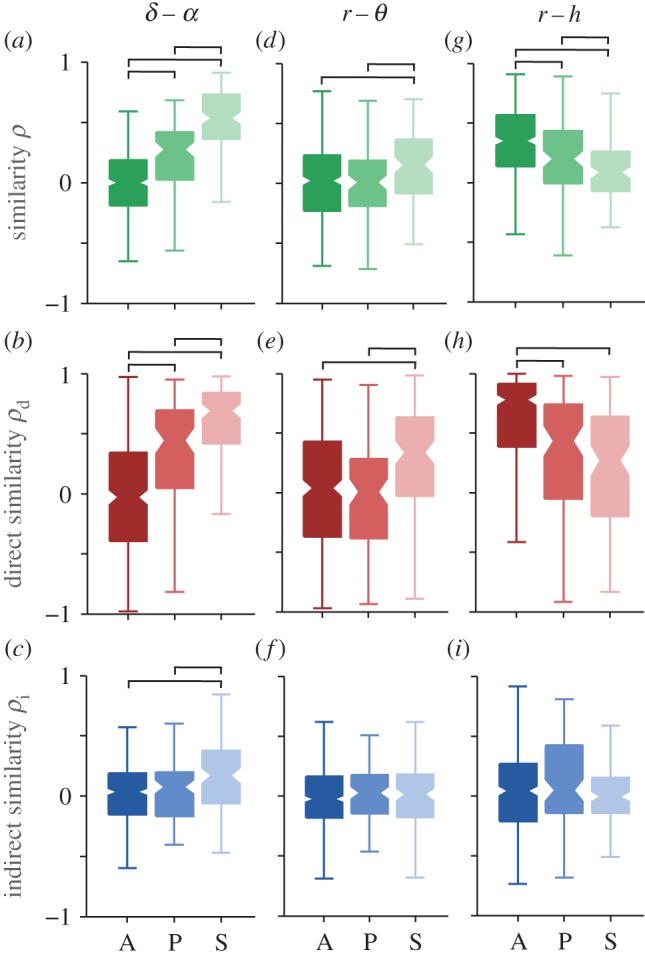
Influence of anaesthesia on the form of the coupling functions. The similarity of functional forms is presented as the correlation coefficient *ρ* for the net ((*a*), (*d*) and (*g*)), the direct ((*b*), (*e*) and (*h*)) and the indirect coupling functions ((*c*), (*f*) and (*i*)). The columns correspond to the three coupling relationships: (*a*–*c*) *δ*–*α*; (*d*–*f*) *r*–*θ* and (*g*–*i*) *r*–*h*. The inter-subject similarity correlation boxplots are shown for the awake (A), propofol (P) and sevoflurane (S) states as indicated on the abscissa. The line connectors on the tops of individual panels indicate cases where the difference between two boxplot distributions was statistically significant. (Online version in colour.)

The other useful characterization of coupling functions is their functional form. It defines the functional law or mechanism and it is a specific feature of coupling functions. To quantify the forms of a given coupling relationship, we use a correlation measure that quantifies the similarity of the forms of two coupling functions, irrespective of their coupling strengths [20]. If the similarities of form for between the intersubject pairs for some interaction is high enough, it means that there exists a common deterministic functional form which underlies the mechanisms of that interaction. From the coupling decomposition model, we can investigate, separately, the similarity of form for each individual component of the coupling functions (figure 6).

The similarity in form of the delta–alpha coupling functions is shown in figure 6*a*–*c*. There is a large difference due to anaesthesia in the net and direct similarity (figure 6*a*,*b*), while the indirect similarity is different only for sevoflurane (figure 6*c*). The similarity of form is especially high for the direct component, while very low for the indirect component. The respiration–theta interaction had relatively small similarity of its functional forms, and there is only a small significant increase for sevoflurane in the net and direct similarities (figure 6*d*,*e*). The respiration-heart interaction also had all the significant differences seen in the net coupling, but now decreased with anaesthesia (figure 6*g*). The similarity of these interactions is mostly due to the high direct similarity (figure 6*h*), where awake is different from when under the two anaesthetics. We note that *anaesthesia had opposite effects* on the similarity of the functional forms for delta–alpha and respiration-heart—cf. figure 6*a*,*g*. This quantitative description is consistent with the observations of the coupling functions made in figure 4.

### (d) The effect of anaesthesia on the noise strength

**Figure 7.**
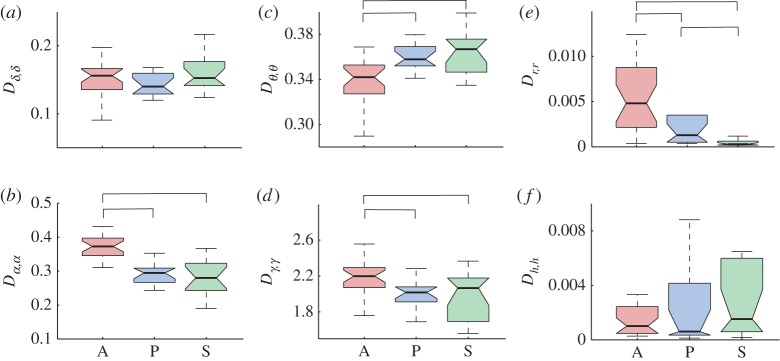
Anaesthesia-induced changes in noise strength. Each boxplot shows the group distribution of all subjects' noise strengths for a specific oscillation interval during the three awakeness states A, P and S. Noise strengths are shown (*a*) for the *δ* oscillation interval, (*b*) *α*, (*c*) *θ*, (*d*) *γ*, (*e*) *r* and (*f*) *h*. The line connectors on the tops of individual panels indicate cases where the difference between two boxplot distributions was statistically significant. (Online version in colour.)

Dynamical Bayesian inference can decompose the dynamics into two parts: what is believed to be the deterministic part of the model; and a part originating from random (white) noise perturbations. The noise strength represents the level of random fluctuations relative to the frequency of the oscillation and its interactions with the other oscillations considered. So we also investigated whether and how anaesthesia affects the noise strength **D** of the brain and cardio-respiratory oscillations, with results as shown in figure 7. Correlated noise strengths, e.g. *D*_*α*,*δ*_ were found to be very small and not statistically different between the awakeness states, so they are not reported. Also, the noise strength for each of the intervals had (qualitatively) the same statistical difference when coupling was investigated with different intervals, e.g. *D*_*α*,*α*_ figure 7*b* was the same whether *δ*−*α* interactions or *α*−*γ* interactions were inferred.

The results in figure 7 demonstrate that the noise strength for some rhythms was unaffected by anaesthesia, *D*_*δ*,*δ*_ in figure 7*a* and *D*_*h*,*h*_ in figure 7*f*; for other rhythms anaesthesia made a significant difference, either increasing like *D*_*θ*,*θ*_ in figure 7*c*, or decreasing like *D*_*α*,*α*_ in figure 7*b* and *D*_*γ*,*γ*_ in figure 7*d*, with anaesthesia relative to the awake state; or the result was statistically different in all three states, like *D*_*r*,*r*_ in figure 7*e*.

## 4. Discussion

The present investigation relies on three complementary factors: (i) general anaesthesia under either intravenous and inhalational anaesthetics; (ii) the novel methodology of cross-frequency *coupling functions* to probe interaction mechanisms directly; and (iii) assessment of the combined dynamics and interactions of the *cortical, respiratory* and *cardiac* oscillations. We have thus been able to analyse the coupling functions between brain activity, which involves information processing and control of the human body, on the one side, and the cardiorespiratory systems, which take care of energy transport and the supplies of nutrients and oxygen, on the other.

While interactions have already been studied in lizards [52], mice [53], rats [15] and dogs [54], here we report the first insights into cardio-respiratory-cortical interactions in humans, in both the awake and anaesthetized states. Moreover, our extension of cross-frequency coupling to include the analysis of coupling functions has allowed us to investigate the interactions in greater depth by introducing the notion of the functional *form*, which represents a new dimension in the analysis of neuronal effective interactions. Thus, we have been able to present two quantitative dynamical properties of the phase interactions: the coupling strength and the form of the coupling function. The functional description of the couplings has enabled us to propose a coupling decomposition model that reveals the separate contributions, in turn providing deeper insight into the causality within a coupling. The model was strongly supported by the results (figures 5 and 6) where the effect of anaesthesia often differed for the individual coupling components.

Coupling functions describe the underlying mechanisms that gives rise to the qualitative states of the interacting systems, e.g. the phase synchronization state which is of great importance in neuronal [37] and cardiorespiratory [20,29] interactions. By knowing the form of the coupling function, one can *predict* the occurrence of phase synchronization for given parameters [16]. Although the discussion was only for large-scale cross-frequency couplings, the coupling functions presented have wide implications at different scales and levels of the heavily connected brain network [35]. Thus one can also describe the functional form of the edges, and can use the coupling decomposition model to investigate the separate contributions from the nodes.

One of the most prominent coupling relationships we identified is delta–alpha. It reflects how delta activity, associated with deep dreamless sleep [55], influences the alpha oscillations which are said to reduce the information processing [55,56] and play a key role in consciousness [57,58]. During the maintenance of general anaesthesia, the alpha and delta activities were increased [2,59]. The delta–alpha coupling has been linked to the coding mechanism of feedback valence information [60]. Even though the anaesthetized state differs from sleep and from the resting state generally, a strong delta–alpha link was observed during non-REM sleep [30] and recently it was suggested that delta–alpha coupling is mostly located within the frontal and the parieto-occipital regions when it is stronger during the eyes-closed state [13]. Our results are consistent with, and further extend and deepen, these findings. Namely, the form of the delta–alpha coupling functions (figures 3 and 6) indicates that the influence is direct modulation from delta to alpha, where the couplings are significantly stronger in anaesthesia than when awake. This shows that, once the subject is anaesthetized, delta activity influences the alpha oscillations by contributing to the reduction of information processing and integration.

Gamma activity, associated with attention, memory and sensory processing, is known to decrease in anaesthesia [61]. In seeking to reveal the underlying mechanisms, we identified two significant couplings: theta–gamma and alpha–gamma. They have been widely studied already, mostly with phase-to-power cross-frequency couplings and higher gamma intervals, and various functional roles have been attributed to them in different states and tasks [62,63]. It has been suggested that theta–gamma coupling plays a prominent role in memory tasks, whereas alpha–gamma interactions are more important for attention processing [64]. The coupling function analysis (figure 4) indicated that these two couplings are affected differently by anaesthesia. Namely, propofol decreased and sevoflurane increased the theta–gamma coupling, while both anaesthetics increased the alpha–gamma coupling (figures 4 and 5). These two couplings evidently have different functional mechanisms. The theta–gamma couplings in anaesthesia result from the direct influence of theta on gamma, while alpha–gamma is dominantly an indirect coupling, implying that there might be a third process which influences both of the oscillations.

We extended the analysis of cross-frequency neuronal couplings to include the interactions of two important parts of the cardiovascular system—the respiration and the heart [4,15]. We identified a coupling function from respiration to theta oscillations. The coupling function was of complex form, with strong direct component and relatively low intensity. The respiration–theta coupling was affected more by the sevoflurane than the propofol anaesthesia.

Of special interest are the *brain–heart interactions* as they have been linked to cardiac arrhythmias, psychophysiological coordination and vascular dementia [65–67]. Our analysis identified a coupling function from the heart to the brain theta oscillations. The form of the coupling function was relatively complex, its intensity was not very high, and the influence was predominantly with a direct component from the heart to the theta oscillations. This coupling function was not greatly affected by the onset of anaesthesia. The origin of the cardiac–theta couplings could be linked to the haemodynamic function of the heart in providing blood, together with oxygen and other metabolic substances, to the brain. Astrocytes and other glial cells might be responsible for mediation of these processes on the neural level [68,69].

The cardiorespiratory coupling function has been extensively studied [19–21] and its direct coupling component [21] and phase resetting curve [20] have been associated with respiratory sinus arrythmia. The functional connectivity of cardiorespiratory interactions was affected in different ways by propofol and sevoflurane anaesthesia [29]. Interestingly, we found that the effect of anaesthesia on the cardiorespiratory coupling functions showed that the coupling strength increased with anaesthesia, whereas the similarity of form decreased (cf. figures 5*m* and 6*g*). This indicates that the inter-subject similarity of forms becomes more varied with anaesthesia, while maintaining stable and strong interactions—perhaps reflecting the chronotaxic nature of the cardiorespiratory interactions [70].

These alterations of the cardiorespiratory coupling functions and their links to the theta brain oscillations (figures 5 and 6) may reflect partially the onset of analgesia and the reduced perception of pain [31,32], with possible links to consciousness. Therefore, such results could have implications for the quest of quantifying analgesia in the absence of consciousness [71,72].

The noise strength analysis in figure 7 shows that anaesthesia changes, not only the deterministic couplings, but also some of the random fluctuations acting on the oscillations. The decrease of the noise level in *α*, *γ* and respiratory oscillations (figure 7*b*,*d*,*e*) might be a consequence of the higher determinism associated with the onset of anaesthesia which induces, e.g. order, coupling and coherence of the oscillations ([2,15] and figure 5). More puzzling is the result that the noise strength for *θ* oscillations increased with anaesthesia, figure 7*c*. It may perhaps be linked to the origin of the *θ* oscillation and its role in the hippocampus [73]. These results are intriguing and invite further investigation using dynamical Bayesian inference, which has clearly demonstrated its potential for studies of this kind as well as for the analysis of (biological) experiments of a stochastic nature quite generally.

Unconsciousness is the most striking change in the state of a subject when general anaesthesia occurs [1,2,74]. The transition to unconsciousness and back can be traced through assessment of the cognitive EEG dynamics [9,10,75] and the recovery of consciousness has been found to differ in elderly subjects [76]. It has been noted that the standard clinical assessments of consciousness (motor, verbal and eye-opening responses [77]) are not sufficient and that there is a need for techniques which also assess the function and effective connectivity [1]. Our statistical comparisons of coupling functions in the awake and anaesthetized states demonstrate that there are significant differences, especially for the delta–alpha and alpha–gamma couplings (figures 3, 5 and 6). These coupling-induced changes of the phase advanced/delayed oscillations alter the attention and memory processes, and suppress information integration which is known for mediating the unconscious state [1].

The roles of propofol and sevoflurane in the induction of unconsciousness as a common mechanism was studied and power differences were outlined [78,75]. Our coupling function results have revealed that these anaesthetics often exhibit similar functional forms, perhaps implying similar mechanisms (figures 3 and 4), but that there are some quantitative differences (figures 5 and 6). In general, we observe similar forms of coupling function, but the strength and effect were significantly stronger for sevoflurane. This could be on account of the doses used. It is also possible that the molecular and neuronal processes associated with propofol and sevoflurane are largely similar, perhaps because both act on the same receptor (e.g. GABA_*A*_) but that there are minor differences in relation to the potassium channels affected [1,5,6].

In conclusion, coupling functions have enabled us to unveil a new perspective on how the neurophysiological mechanisms are affected by general anaesthesia. This initial application has been in a sense overwhelming in that we have identified six important and very illuminating coupling relationships. This was partly because we analysed not only neuronal oscillations, but also how the latter are affected by cardiorespiratory activity. The work has opened the door to a host of new questions and problems needing to be tackled. For example, can one apply coupling function analysis to assess spatial neuronal couplings using additional EEG electrodes, perhaps using different anaesthetics? The possibility of following time-evolving dynamics could lead to new insights based on studies of how the evolution of the coupling functions mechanisms lead to unconsciousness. Coupling functions can also be used to study the mechanisms of other neurophysiological perturbations, as well as to revisit known problems, states and diseases in order to reveal the underlying functional mechanisms. Needless to say, the findings and the methodology of this work also have wide implications for coupled oscillators in general, with the possibility of biomimetic [79] solutions to a diversity of difficult problems [80,23].
